# Passive immunization does not provide protection against experimental infection with *Mycoplasma haemofelis*

**DOI:** 10.1186/s13567-016-0361-x

**Published:** 2016-08-05

**Authors:** Sarah Sugiarto, Andrea M. Spiri, Barbara Riond, Marilisa Novacco, Angelina Oestmann, Luisa H. Monteiro de Miranda, Marina L. Meli, Felicitas S. Boretti, Regina Hofmann-Lehmann, Barbara Willi

**Affiliations:** 1 Clinical Laboratory, Vetsuisse Faculty, University of Zurich, Winterthurerstr. 260, 8057 Zurich, Switzerland; 2 Center for Clinical Studies, Vetsuisse Faculty, University of Zurich, Winterthurerstr. 260, 8057 Zurich, Switzerland; 3 Clinic for Small Animal Internal Medicine, Vetsuisse Faculty, University of Zurich, Winterthurerstr. 260, 8057 Zurich, Switzerland; 4 Laboratory of Clinical Research on Dermatozoonosis in Domestic Animals, National Institute of Infectiology, Oswaldo Cruz Foundation, Rio de Janeiro, Brazil

## Abstract

*Mycoplasma haemofelis* (*Mhf*) is the most pathogenic feline hemotropic mycoplasma. Cats infected with *Mhf* that clear bacteremia are protected from *Mhf* reinfection, but the mechanisms of protective immunity are unresolved. In the present study we investigated whether the passive transfer of antibodies from *Mhf*-recovered cats to naïve recipient cats provided protection against bacteremia and clinical disease following homologous challenge with *Mhf*; moreover, we characterized the immune response in the recipient cats. Ten specified pathogen-free (SPF) cats were transfused with pooled plasma from cats that had cleared *Mhf* bacteremia; five control cats received plasma from naïve SPF cats. After homologous challenge with *Mhf*, cats were monitored for 100 days using quantitative PCR, hematology, blood biochemistry, Coombs testing, flow cytometry, DnaK ELISA, and red blood cell (RBC) osmotic fragility (OF) measurement. Passively immunized cats were not protected against *Mhf* infection but, compared to control cats, showed significantly higher RBC OF and B lymphocyte (CD45R/B220^+^) counts and occasionally higher lymphocyte, monocyte and activated CD4^+^ T lymphocyte (CD4^+^CD25^+^) counts; they also showed higher bilirubin, total protein and globulin levels compared to those of control cats. At times of peak bacteremia, a decrease in eosinophils and lymphocytes, as well as subsets thereof (B lymphocytes and CD5^+^, CD4^+^ and CD8^+^ T lymphocytes), and an increase in monocytes were particularly significant in the passively immunized cats. In conclusion, passive immunization does not prevent bacteremia and clinical disease following homologous challenge with *Mhf,* but enhances RBC osmotic fragility and induces a pronounced immune response.

**Electronic supplementary material:**

The online version of this article (doi:10.1186/s13567-016-0361-x) contains supplementary material, which is available to authorized users.

## Introduction

Hemotropic mycoplasmas (hemoplasmas) are non-cultivable epierythrocytic bacteria that infect a variety of mammalian species worldwide [1]. In recent years, hemoplasmas have attracted scientific attention due to their host diversity and pathogenic potential [1]. The main pathogenic feature of hemoplasmas is hemolysis, and clinical signs such as lethargy, anorexia, pale mucosal membranes, pyrexia, jaundice and pigmenturia may be present in severely affected animals [1]. Reports of hemoplasma infections in humans emphasize the need to characterize these agents in more detail [2–7]. Feline hemoplasmas can thereby serve as a model because of their extensive molecular and clinical characterization within this group of organisms.

Feline hemoplasmas comprise at least three different species: *Mycoplasma haemofelis* (*Mhf*), “*Candidatus* Mycoplasma haemominutum” (*CMhm*) and “*Candidatus* Mycoplasma turicensis” (*CMt*) [8–10]. Concurrent infections with two or three hemoplasma species have been documented [11–14], suggesting that no immunological cross-protection exists between the feline hemoplasmas, which has recently been experimentally confirmed [15]. *Mhf* is the most pathogenic of the three feline hemoplasma species and can induce severe hemolytic anemia, which is potentially fatal if left untreated. In contrast, the other two feline hemoplasmas may induce mild anemia, and the infection often remains subclinical [16].

The natural route of hemoplasma transmission between cats is still unresolved, but aggressive interactions and blood-sucking arthropods have mainly been implicated [17–19]. For experimental transmission, the intraperitoneal, intravenous or subcutaneous inoculation of hemoplasma-containing blood has been successful [10, 19–21]. Recently, a low-dose infection model for *Mhf* that aimed to more accurately mirror the natural transmission of hemoplasmas was developed [22].

Different antibiotic regimens reduce hemoplasma blood loads and alleviate clinical signs but, so far, no treatment protocol has successfully and consistently cleared feline hemoplasma infections [21, 23–26]. This limitation emphasizes the need to further investigate protective immune mechanisms against these agents. Recently, cats that were experimentally infected with *Mhf* or *CMt* and overcame bacteremia were shown to be protected from reinfection with the same hemoplasma species [27, 28]. A study by Novacco et al. [27] suggested a significant role for the humoral immune response in protecting against *CMt* reinfection: nine out of the ten cats that were protected from reinfection showed intermediate to high antibody levels against *CMt* before challenge. Furthermore, a transient decrease in antibody levels was observed in the protected cats immediately after attempted reinfection, which could be due to the binding of antibodies to the inoculated antigens. In the early phase after re-challenge, compared with the control group, the protected cats exhibited significantly higher IL-4/IL-12 ratios and CD4^+^ T lymphocyte counts and a pronounced eosinophilia. Therefore, the authors concluded that an early Th2 immune response, prior to the onset of bacteremia, is beneficial for protection against *CMt* reinfection [27]. This result was not found in the study by Hicks et al. [28], where an early increase in the pro-inflammatory cytokines tumor necrosis factor-α (TNF-α) and interleukin-6 (IL-6) was observed in cats protected from *Mhf* reinfection. Furthermore, the immune response seemed to be skewed towards a Th1 response after primary *Mhf* infection, whereas a switch from an initial Th1 to a delayed Th2 response was observed after primary *CMt* infection [27, 28]. These results suggest that cats respond to infection by different feline hemoplasma species with different immune mechanisms.

Important data on the immune response elicited by hemoplasma infection have been provided by previous studies [27, 28], but the mechanisms that confer protection against re-infection have yet to be clarified. Passive immunization transfers humoral immunity to a non-immune individual in the form of antibodies and allows the protective role of antibodies in the absence of cellular immune mechanisms to be assessed. The present study aimed to investigate whether the passive transfer of antibodies from *Mhf*-recovered to naïve recipient cats induced partial or complete protection against bacteremia and clinical disease following homologous challenge with *Mhf*. Different parameters addressing the humoral and cellular immune response were monitored in passively immunized and control cats.

## Materials and methods

### Animals

In the main experiment, 15 4-month-old male specified pathogen-free (SPF) cats from Liberty Research, Inc. (Waverly, New York, USA) were used as recipient cats. Five adult SPF cats served as blood donors: three of these cats had overcome a previous *Mhf* infection (HBU2, HBZ2 and HCD2) [28], while two cats were naïve adult SPF cats (GCN5 and JCR4). Moreover, in a pre-experiment, a 2-year-old male castrated SPF cat (HCC4) was used. All cats were housed in groups in a confined university facility under ethologically and hygienically ideal conditions [29]. All experiments were approved by the veterinary office of the Canton of Zurich (TVB 92/2014) and were conducted in accordance with Swiss laws.

Fifty days prior to plasma transfusion and experimental infection, the SPF status of all the cats was confirmed by testing for the absence of infection with feline hemoplasmas, feline calicivirus, feline herpesvirus-1, feline coronavirus, feline leukemia and feline immunodeficiency virus, as well as *Bartonella henselae* and *Chlamydia felis*, as recently described [19, 30]; the absence of *Mhf* infection was again confirmed by real-time TaqMan^®^ quantitative (q)PCR [12] on day 0 prior to plasma transfusion and *Mhf* inoculation. All donor cats and the cat from the pre-experiment were blood-typed using a commercial immunochromatography technique (Feline Lab Test A + B, Alvedia, France). The recipient cats had been blood-typed by Liberty Research, Inc. (Waverly). A standard saline-agglutination cross-matching procedure was performed to ascertain the compatibility of transfusion [31].

### Plasma preparation and transfusion

For passive immunization, a total of 118 mL of plasma was collected from the three cats that had overcome previous *Mhf* infection (HBU2, HBZ2 and HCD2) [28]. The plasma was collected from these cats on day 538 post *Mhf* inoculation. HBU2 had cleared the infection without treatment, whereas HBZ2 and HCD2 had received antibiotic treatment to clear the infection (HBZ2: 10 mg/kg/day doxycycline for 44 days; HCD2: 10 mg/kg/day doxycycline for 27 days, followed by 2 mg/kg/day marbofloxacin for 23 days); in the latter two cats, antibiotic treatment was stopped 59–129 days prior to plasma collection. All cats tested negative for *Mhf* for at least eight consecutive weeks following antibiotic treatment and prior to the plasma collection, as determined using weekly collected blood samples that were analyzed in triplicate with a *Mhf*-specific qPCR assay [12]. For plasma transfusion of the control cats, a total of 70 mL of plasma from two adult naïve SPF cats (GCN5 and JCR4) was collected. Prior to plasma collection, the two cats were confirmed to be negative for all three feline hemoplasmas using qPCR [12] and to be free from all commonly known feline pathogens, as described [19].

For plasma preparation, whole blood was collected from the donor cats from the jugular vein under short-duration general anesthesia (10 mg/kg ketamine, Narketan^®^, Vétoquinol AG, Belp, Switzerland; 0.1 mg/kg midazolam, Dormicum^®^, Roche Pharma AG, Reinach, Switzerland); the blood was anti-coagulated with acid-citrate-dextrose (ACD) to a final concentration of 3.5%. Plasma was separated from RBCs by centrifugation at 3000*g* for 10 min and stored at −80 °C until use. Prior to transfusion, the plasma was thawed, pooled (pool A: plasma from the *Mhf*-recovered cats HBU2, HBZ2 and HCD2; pool B: plasma from the naïve SPF cats GCN5 and JCR4), and filtered through a 0.22 µm pore-size filter (JET BIOFIL, Madrid, Spain), and 9 mL aliquots were prepared. Before freezing and after thawing and filtration, the plasma was tested in triplicate with a *Mhf*-specific qPCR assay for the absence of *Mhf* organisms [12].

For the plasma transfusion, all recipient cats were put under short-duration general anesthesia (10 mg/kg ketamine, Narketan^®^, Vétoquinol AG; 0.1 mg/kg midazolam, Dormicum^®^, Roche Pharma AG) and 9 mL of whole blood was collected for baseline analysis and to prevent circulatory volume overload. The plasma was pre-warmed to 38 °C and intravenously administered over a duration of 30 min using standard blood transfusion sets (HEMO-NATE^®^, 18 Micron, Utah Medical Products, Inc., Utah, USA). During transfusion, the cats were closely monitored for transfusion reactions.

### Experimental design

In the pre-experiment, an adult naïve SPF cat (HCC4) was intravenously transfused with a 10 mL aliquot of plasma pool A that was later used for the passive immunization of the cats in group A. After the transfusion, weekly blood samples were collected from the cat for 23 weeks and analyzed using *Mhf*-specific qPCR [12].

For the main experiment, the recipient cats were assigned to two groups: group A (*n* = 10, passive immunization, cats DHR1, DHR2, JHW1, JHW2, JHW4, JHW5, JHX1, JHX2, JHZ1 and JIC1) and group B (*n* = 5, control group, cats DHP1, DHT1, JHV1, JHW3 and JIC2). The cats of each group were housed together during and after the end of the study period. On day 0, plasma transfusion was performed, and the cats were experimentally inoculated with *Mhf*. For the passive immunization, each cat in group A was transfused with a 9 mL aliquot of plasma pool A. Each cat in group B was transfused with a 9 mL aliquot of plasma pool B. Ten minutes after the completion of the transfusion, all cats in groups A and B were inoculated with *Mhf* by a subcutaneous injection of 10 µL of 20% dimethyl sulfoxide (DMSO)-preserved blood containing 10^3^ copies of *Mhf* diluted with 90 µL of phosphate-buffered saline (PBS), as previously described [22]. An aliquot of the same *Mhf* inoculum was used to infect the recipient cats in this study that had been used to infect the plasma donor cats (HBU2, HBZ2 and HCD2) in a previous study [28]. Clinical condition, body temperature and body weight were recorded, and blood samples collected prior to plasma transfusion and *Mhf* inoculation (day 0), twice weekly post inoculation (pi) until week 7 and weekly thereafter up to day 100 pi.; an additional blood collection to measure red blood cell (RBC) osmotic fragility (OF) was performed on day 179 pi because RBC OF was still increased compared with baseline values in several cats of group A and B at day 100 pi. All samples were collected without anesthesia, with the exception of the samples collected at day 0. Samples for PCR analysis and serology were stored at −80 °C until analysis. Flow cytometry, hematology, blood biochemistry, Coombs tests and RBC OF assays were carried out within 4 h of blood collection.

### Hematology and blood biochemistry

Hematological parameters were determined on day 0, twice weekly pi until week 7 and weekly thereafter up to day 100 pi on a Sysmex XT-2000iV instrument (Sysmex Corporation, Kobe, Japan), as previously described [32]. White blood cell differential analysis was confirmed using manual evaluation of Wright-Giemsa stained blood smears. Severity of anemia was defined as mild (25–33%), moderate (15–25%) and severe (<15%). Bilirubin, total protein and albumin concentrations were measured on day 0, on days 3 and 7 pi and weekly thereafter until day 100 pi using a Cobas Integra 800 system (Roche Diagnostics, Rotkreuz, Switzerland). Globulin values were calculated by subtracting the albumin value from the total protein concentration. Reference intervals (mean ± standard deviation, SD) for PCV and total protein concentrations for kittens of 16 and 30 weeks of age were obtained from the literature [33]. Because bilirubin concentrations reach adult values after 1 week of age [33], the laboratory’s device-specific reference interval was used for this parameter.

### RBC OF and direct Coombs testing

RBC OF was determined on day 0 and on days 21, 100 and 179 pi using a published protocol [10]. The reference interval was determined in nine healthy cats during a previous study (50% hemolysis in NaCl 0.50–0.57% w/v) [10]. Direct Coombs tests were performed on day 0 and on days 21 and 100 pi. RBCs from EDTA-anticoagulated blood samples were washed, diluted in NaCl 0.9% w/v solution and incubated for 1 h at 37 °C with feline antiglobulin reagent (MP Biomedicals, LLC., Solon, Ohio, USA) in dilutions ranging from 1:2 to 1:10240, as described [10]. Agglutination in dilutions of ≥1:8 was defined as positive.

### Quantification of bacterial loads

Quantification of *Mhf* blood loads by qPCR was performed on day 0, twice weekly pi until week 7 and weekly thereafter up to day 100 pi. Total nucleic acid (TNA) was extracted from 100 μL of EDTA anti-coagulated blood using the MagNa Pure LC (Roche Diagnostics AG, Rotkreuz, Switzerland) and the MagNa Pure LC TNA Isolation Kit (Roche Diagnostics) following the manufacturer’s instructions. TNA was eluted in 100 µL elution buffer and stored at −80 °C until use. With each batch of extraction, a negative control consisting of 200 µL of phosphate-buffered saline (PBS) was used to monitor for cross-contamination. All TNA samples were tested with a *Mhf*-specific qPCR assay to detect and quantify *Mhf* as previously described [12]. For absolute quantification, tenfold serial dilutions of a standard plasmid were used as described [12]. Positive and negative controls were included in each PCR run.

### Serology

Antibodies against the recombinant DnaK protein of *Mhf* were measured on day 0, on days 3 and 7 pi and weekly thereafter until day 100 pi using a previously described ELISA [20]. The serum was diluted 1:200, and 50 ng of recombinant protein per well was used as described [20]. All samples collected from one cat were measured on the same plate, and all plates were antigen coated within the same batch. Absorbance was measured at a wavelength of 415 nm (OD_415_) using a SpectraMax Plus 348 microplate spectrophotometer (Molecular Devices, Sunnyvale, CA, USA). An OD_415_ ≤ 0.33 was defined as negative based on DnaK ELISA results obtained from 20 SPF cats, as described [20].

### Flow cytometry

Flow cytometric analysis was performed on day 0 and on days 3, 7, 14, 21, 28, 42, 56, 70 and 98 pi. Three different staining combinations of primary antibodies were used: (1) Fluorescein isothiocyanate (FITC)-conjugated mouse anti-feline CD5 antibody (f43, Southern Biotech, Allschwil, Switzerland); (2) an R-phycoerythrin (RPE)-conjugated mouse anti-feline CD4 antibody (Vpg34, AbD Serotec, Puchheim, Germany) and a FITC-conjugated mouse anti-feline CD25 antibody; and (3) a FITC-conjugated mouse anti-feline CD8 antibody (fCD8, Southern Biotech) and a RPE-conjugated rat anti-mouse CD45R/B220 antibody (RA3-6B2, AbD Serotec). CD5 is a marker for feline T lymphocytes [34], and CD4^+^- and CD8^+^-positive T lymphocytes represent helper and cytotoxic T lymphocytes, respectively [35]. CD4^+^CD25^+^ T lymphocytes represent activated CD4^+^ T lymphocytes, and CD45R/B220 antibody was used to identify B lymphocytes [36, 37].

Prior to the start of the experiment, the antibodies were titrated for optimal dilution as follows: CD4 and CD8, 1:50 dilution; CD25 and CD5, 1:100 dilution; and CD45R/B220, undiluted. A 50 µL aliquot of EDTA anti-coagulated blood was incubated with 5 µL of the aforementioned antibodies. To rule out non-specific binding, isotype control antibodies (RPE-conjugated Mouse IgG1 and FITC-conjugated Mouse IgG1, AbD Serotec) and unstained blood samples from each cat were used as negative controls. Blood samples were stained according to published protocols [27, 28].

Flow cytometry was performed using a guava easyCyte™ 8HT Flow Cytometer (Millipore, Darmstadt, Germany) using the GuavaSoft 2.5 software. Gates representing the lymphocyte population were set based on forward and side scatter, and 10 000 events were acquired for each sample. The absolute number of each lymphocyte subset was calculated by multiplying the absolute lymphocyte number from hematology by the subset percentage as previously published [38].

### Statistics

Up to 22 different parameters per cat were statistically analyzed using Analyse-it^®^ for Microsoft Excel version 1.0.5.0 (Analyse-it Software, Ltd., Leeds, UK) and GraphPad Prism 5.03 (GraphPad Software, Inc., CA, USA). The Mann–Whitney U test (p_MWU_) was used to compare the two groups A and B at each time point. One cat (JHW3) in group B stayed PCR-negative and seronegative throughout the 100-day study period. To investigate whether significant differences observed between groups A and B were related to the inclusion of this cat, the Mann–Whitney U tests were repeated without the data of this cat for all significant parameters. The results from these analyses were reported if they differed from the original results including cat JHW3. Friedman’s test (p_F_) followed by Dunn’s post test (p_D_) was used to analyze the parameters over time when more than two time points were considered. Multiplicity adjusted *P* values for each comparison in a family of comparisons were computed. Fisher’s exact test was used to determine significant differences of proportions. *P* values <0.05 were considered significant.

## Results

All donor cats, the cat from the pre-experiment and all recipient cats were confirmed to be blood type A. Cross-matching revealed no incompatibilities between the donor cats, the cat of the pre-experiment and the recipient cats. All plasma samples used for transfusion tested *Mhf* PCR-negative.

### Pre-experiment

The adult cat (HCC4) transfused with an aliquot of plasma pool A during the pre-experiment tested *Mhf* PCR-negative in each of the samples collected during the 23 weeks after the transfusion (data not shown). The cat stayed clinically healthy throughout the entire experiment.

### Passive immunization does not provide protection against *Mhf* bacteremia

**Figure 1:**
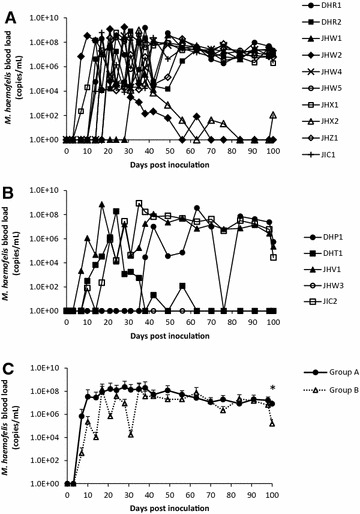
***Mhf*** **blood loads in passively immunized and control cats after** ***Mhf*** **infection.** *Mhf* blood loads (y-axis, as log copies/mL of blood) of individual cats in group A (**A**) and B (**B**) and mean (+SD) *Mhf* blood loads in group A and B (**C**) are shown during the 100 days pi. Because of the logarithmic scale, only the positive SD is shown in (C). All cats were subcutaneously inoculated with *Mhf* at day 0. Significant differences between groups are indicated with asterisks (p_MWU_ < 0.05). Cat JHW3 in group B stayed PCR-negative throughout the 100-day follow-up period but turned PCR-positive at day 154 pi (not shown).

Prior to plasma transfusion and experimental infection (day 0), all cats in both groups tested *Mhf* PCR-negative. All cats in groups A and B became *Mhf* PCR-positive within 7–31 days and 7–38 days pi, respectively (Figures 1A and B), except for one cat (JHW3) in group B. Cat JHW3 remained PCR-negative throughout the 100-day follow-up period (Figure 1B) but became *Mhf* PCR-positive after the end of the experiment (day 154 pi, data not shown). Peak hemoplasma loads (group A: 2.3 × 10^8^–1.7 × 10^9^ copies/mL blood; group B 1.8 × 10^8^–8.6 × 10^8^ copies/mL blood) were reached between days 17 and 49 pi in group A and days 17 and 63 pi in group B. There was no significant difference in *Mhf* blood loads between the two groups during the 100-day follow-up period, except for day 100 pi, when group A showed significantly higher *Mhf* blood loads (p_MWU_ = 0.0280; Figure 1C); this difference was not significant when the PCR-negative cat JHW3 in group B was excluded from the analysis (p_MWU_ = 0.0759).

### Anti-DnaK antibody levels were higher in passively immunized cats than in control cats in the first days after plasma transfusion

**Figure 2:**
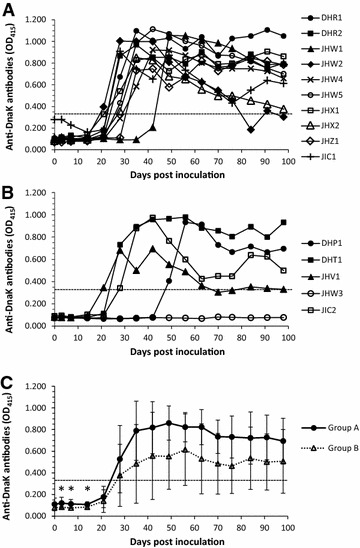
**Anti-DnaK antibody response in passively immunized and control cats.** Anti-DnaK antibody levels (y-axis, as OD_415_ values) of individual cats in group A (**A**) and B (**B**) and mean (±SD) anti-DnaK antibodies in group A and B (**C**) during the 100 days pi. All cats were subcutaneously inoculated with *Mhf* at day 0. An OD_415_ value of 0.33 is indicated by a dotted line and represents the threshold for seropositivity. Significant differences between groups are indicated with asterisks (p_MWU_ < 0.05).

The cats in both groups tested negative in the DnaK ELISA (defined as an OD_415_ ≤ 0.33) prior to the plasma transfusion (Figures 2A and B). After passive immunization and subsequent infection, group A cats showed slightly but significantly higher DnaK ELISA OD_415_ values at days 3, 7 and 14 pi compared with the OD_415_ values of cats in group B (p_MWU_ < 0.05; Figure 2C). From day 21 until day 100 pi, no significant difference in the DnaK ELISA OD_415_ values was evident between the two groups. When cat JHW3 was excluded from the analyses, the anti-DnaK antibody level in cats in group A was still significantly higher early after passive immunization (days 3 and 7 pi, P_MWU_ < 0.05), but not at day 14 pi and thereafter until day 100 pi when compared to cats in group B. There was no significant difference in the time of seroconversion (OD_415_ > 0.33) between the two groups; cats in both groups, A and B, seroconverted between days 21 and 49 pi, with the exception of cat JHW3 (group B), which did not develop anti-*Mhf* DnaK antibodies throughout the study (Figure 2B). Two other cats (JHW2, group A, and JHV1, group B) were seronegative (OD_415_ ≤ 0.33) by the end of the experiment (Figure 2B); one of these cats (JHW2) was treated with doxycycline between days 28 to 42 pi because of a pronounced decrease in packed cell volume (PCV) to 21% (day 28 pi, see below).

### No difference in PCV and clinical signs between the passively immunized and control cats

**Figure 3:**
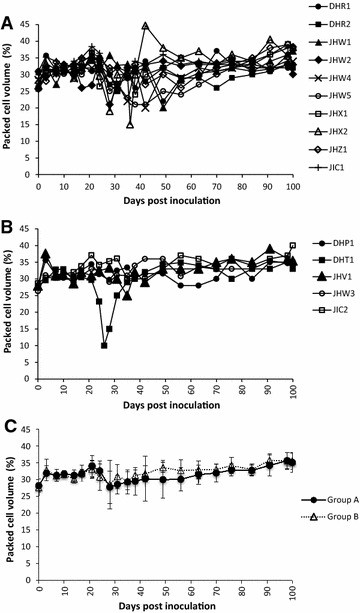
**Course of PCV in passively immunized and control cats.** PCV values (y-axis, as percentage) of individual cats in group A (**A**) and B (**B**) and mean (±SD) PCV values in group A and B (**C**) during the 100 days pi are shown. All cats were subcutaneously inoculated with *Mhf* at day 0. There was a significant change in PCV over time in cats in group A (p_F_ < 0.0001, lower values at days 0, 31, 35 and 56 compared with days 21, 91, 98 and 100 pi, p_D_ < 0.05) and group B (p_F_ = 0.0003, significantly lower values at day 0 compared with days 91, 98 and 100 pi, p_D_ < 0.05).

Seven out of ten cats in group A became moderately anemic (PCV between 15 and 25%) at days 28 to 56 pi (Figure 3A). In group B, one cat showed moderate anemia (PCV 25%) and one cat severe anemia (PCV 10%) at days 35 and 26 pi, respectively (Figure 3B). The lowest PCV observed during the study was found for cat DHT1 (PCV 10%, 26 days pi) in group B. There was no significant difference in the mean PCV values between the two groups (Figure 3C). There was a significant change in PCV over time for both groups (group A, p_F_ < 0.0001; group B, p_F_ = 0.0003); PCV values of the cats were higher towards the end of the study period when compared to the first weeks pi (Figures 3A and B). This finding may be attributed to the increasing age of the cats; reference intervals of PCV (±SD, %) were reported to increase from the age of 16 weeks (34.9 ± 1.1) to 30 weeks (37.1 ± 3.4) [33].

Three cats in group A (cats JHW2, JHW4 and JHZ1) and two cats in group B (cats DHT1 and JHV1) developed elevated body temperature >39.5 °C during the course of the experiment: JHV1 at day 12 pi, JHW4 at day 23 pi, DHT1 at day 26 pi, JHW2 at day 28 pi and JHZ1 at day 48 pi. In addition, apathy, anorexia and weakness were observed in three cats in group A (JHW2 and JIC1 at day 28 pi; and JHX2 at day 36 pi) and in one cat in group B (DHT1 at day 26 pi).

Three cats necessitated oral antibiotic treatment with doxycycline (10 mg/kg/day, Supracycline, Grünenthal GmbH, Mitlödi, Switzerland) for 14–16 days. The decision to treat the cats was based on the presence of severe anemia (group A: DHT1, PCV of 10%, day 26 pi) or a pronounced decrease in PCV within 24 h (JHW2, PCV of 21%, day 28 pi; JHX2, PCV of 15%, day 36 pi). All three cats showed clinical signs (apathy, anorexia, weakness and fever) when the treatment was initiated.

### Higher RBC OF in passively immunized than in control cats

**Figure 4:**
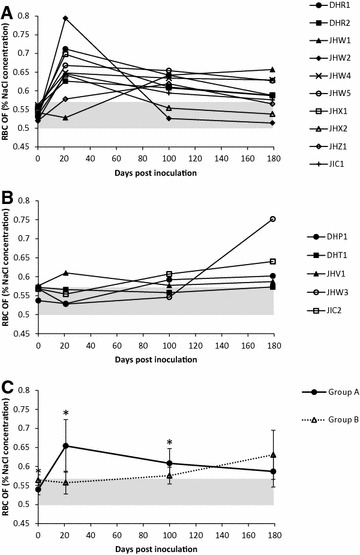
**Course of RBC OF in passively immunized and control cats.** RBC OF (y-axis, as percentage (w/v) NaCl concentration with 50% hemolysis) of individual cats in group A (**A**) and B (**B**) at day 0 and at days 21, 100 and 179 pi and mean (±SD) RBC OF in group A and B (**C**) are shown. All cats were subcutaneously inoculated with *Mhf* at day 0. Significant differences between groups are indicated with asterisks (p_MWU_ < 0.05). The reference interval for RBC OF based on measurements in nine healthy cats is shaded grey (50% hemolysis in NaCl 0.50–0.57% w/v) [10]. RBC OF significantly changed during the course of infection in cats in group A (p_F_ = 0.0004, significantly higher levels at days 21 and 100 pi compared with day 0 pre-inoculation, p_D_ < 0.05), but not in cats in group B.

At day 0, RBC OF was significantly lower in cats in group A when compared to cats in group B (p_MWU_ = 0.0127), but mean RBC OF of both groups remained within the reference interval (group A and B, 50% hemolysis in NaCl 0.54 and 0.56% w/v, respectively; reference interval, 50% hemolysis in NaCl 0.50–0.57% w/v, Figure 4C). Thereafter, at days 21 and 100 pi, RBC OF was significantly higher in cats in group A compared to cats in group B (p_MWU_ < 0.05; Figure 4C). When cat JHW3 was excluded from the analyses, there was no longer a significant difference between the groups on day 100 pi. Remarkably, compared with baseline values, RBC OF was still increased in several cats in groups A and B at day 100 pi (Figures 4A and B ); therefore, one further measurement was obtained at day 179 pi. RBC OF significantly changed during the course of infection only in cats in group A (p_F_ = 0.0004, Figure 4A). The RBC OF reached maximal values at day 21 pi in eight out of ten animals in group A (range, 50% hemolysis in NaCl 0.63–0.79% w/v) and at day 100 pi in cat JHZ1 (50% hemolysis in NaCl 0.62% w/v) and at day 179 pi in cat JHW1 (50% hemolysis in NaCl 0.66% w/v; Figure 4A). Cat JHW3 in group B, which turned *Mhf* PCR-positive only after the end of the experiment (day 154 pi), showed a pronounced increase in relative RBC OF at the time of *Mhf* bacteremia (50% hemolysis in NaCl 0.75% w/v, day 179 pi; reference interval, 50% hemolysis in NaCl 0.50–0.57% w/v; Figure 4B). All cats in both groups tested negative in the direct Coombs test at all time points (data not shown).

### Decrease in lymphocyte and eosinophil counts and increase in monocyte counts coinciding with maximal Mhf bacteremia

**Figure 5:**
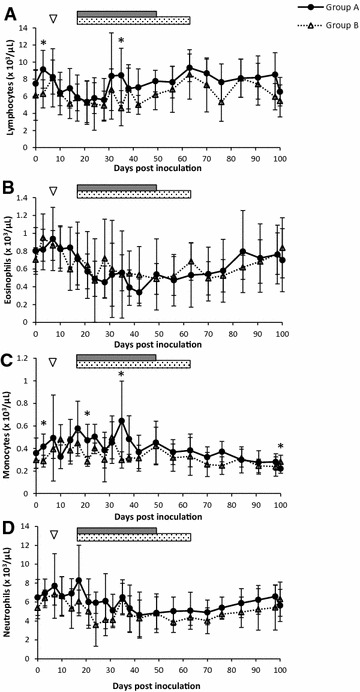
**Lymphocyte, eosinophil, monocyte and neutrophil counts during the course of** ***Mhf*** **infection.** Mean (±SD) absolute cell counts of lymphocytes (**A**), eosinophils (**B**), monocytes (**C**) and neutrophils (**D**) during the 100-day pi period in group A (black dots, solid line) and group B (open triangles, dashed line). Cats were subcutaneously inoculated with *Mhf* at day 0. The onset of bacteremia (day 7 pi for both groups) is indicated with a triangle. Boxes represent the time of peak bacteremia in cats in group A (grey box) and group B (dotted box). Statistically significant differences between groups A and B are indicated with asterisks (p_MWU_ < 0.05). **A** Lymphocyte cell counts changed significantly over time in group A (p_F_ < 0.0001; lower at days 17, 21, 24 and 28 pi compared with days 3, 63 and 70 pi, p_D_ < 0.05) and group B (p_F_ = 0.0033, no significance in the posttest). **B** Eosinophil cell counts changed significantly over time in group A (p_F_ < 0.0001, lower at days 38, 42, 49, 56 and 63 pi compared with day 0 and days 3, 7, 10, 14, 84 and 98 pi, p_D_ < 0.05) and group B (p_F_ = 0.0067, no significance in the posttest). **C** Monocyte cell counts changed significantly over time in group A (p_F_ < 0.0001, higher at days 3, 7, 14, 17, 21, 24, 31, 35, 38 and 49 pi compared with days 84, 91, 98 and 100 pi, p_D_ < 0.05) and group B (p_F_ = 0.0135, no significance in the posttest). **D** Neutrophil cell counts changed over time only in group A (p_F_ = 0.0007, no significance in the posttest).

Cats in both groups showed an increase in leukocyte counts immediately after infection, followed by a significant decrease, with the nadir being reached three to four weeks pi (data not shown). There was no significant difference in leukocyte counts between the two groups during the 100-day follow-up period. The lymphocyte cell counts showed an initial increase followed by a significant decrease, with the nadir reached at day 21 pi, coinciding with the onset of peak bacteremia in cats in group A (p_F_ < 0.0001, Figure 5A); this pattern was less pronounced in cats in group B (p_F_ = 0.0033, Figure 5A). Furthermore, compared with cats in group B, cats in group A had significantly higher blood lymphocyte counts at days 3 and 35 pi (p_MWU_ < 0.05), as well as a tendency for higher counts at day 42 pi (p_MWU_ = 0.0553, Figure 5A). When cat JHW3 was excluded from the analyses, the lymphocyte cell counts tended to be higher in group A compared to group B during the study period, but significance was not achieved. The eosinophils showed a similar pattern to the lymphocyte counts in cats in group A, but the decrease in cell counts was delayed, and the nadir was reached at 6 weeks pi (p_F_ < 0.0001, Figure 5B); again, this pattern was less pronounced in cats in group B (p_F_ = 0.0067, Figure 5B). In contrast, the monocyte counts increased after infection in cats in group A, with maximal values reached at day 35 pi (p_F_ < 0.0001, Figure 5C); again, this pattern was less pronounced in cats in group B (p_F_ = 0.0135, Figure 5C). When compared to the counts in group B, the monocyte counts in group A were higher at most of the time points pi, with significance reached at days 3, 21, 35, and 100 pi (p_MWU_ < 0.05, Figure 5C). The monocyte cell counts were higher in group A compared to group B at most time points pi when cat JHW3 was excluded, with significance reached at days 3 and 21 pi (P_MWU_ < 0.05). The neutrophils significantly changed over time only in cats in group A (p_F_ = 0.0007, Figure 5D). There were no significant differences in the neutrophil counts between the two groups.

When the course of the different white blood cell subsets was compared with the course of PCV and *Mhf* bacteremia, most cats showed a decrease in the leukocyte, lymphocyte, and neutrophil cell counts just prior to the decrease in PCV and the onset of maximal *Mhf* blood loads (data not shown). A similar pattern was also observed for the eosinophils, whereas the monocyte counts peaked at the onset of maximal anemia in most of the cats (data not shown).

### Higher B lymphocytes in passively immunized than in control cats

**Figure 6:**
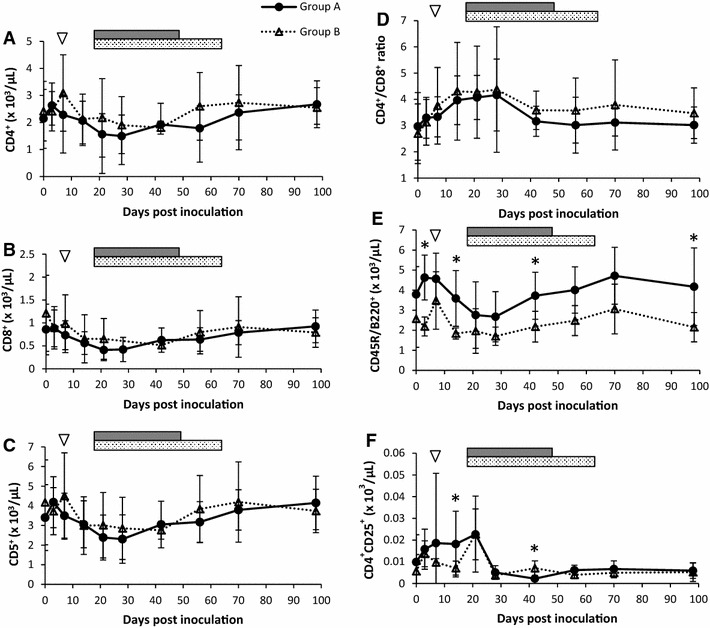
**CD4** ^**+**^ **, CD8** ^**+**^ **and CD5** ^**+**^ **T lymphocytes, B lymphocytes and activated CD4** ^**+**^ **T lymphocytes during the course of** ***Mhf*** **infection.** Mean (±SD) cell counts of the CD4^+^ (**A**), CD8^+^ (**B**) and CD5^+^ T lymphocytes (**C**), CD4^+^/CD8^+^ ratio (**D**), B lymphocytes (CD45R/B220^+^, **E**) and activated CD4^+^ T lymphocytes (CD4^+^CD25^+^, **F**) at day 0 and during the 100-day pi period in group A (black dots, solid line) and group B (open triangles, dashed line). Cats were subcutaneously inoculated with *Mhf* at day 0. The onset of bacteremia in group A and B (day 7 pi, both groups) is indicated with a triangle. Boxes represent the time of peak bacteremia in group A (grey box) and B (dotted box). Statistically significant differences between group A and B are indicated with asterisks (p_MWU_ < 0.05). **A** CD4^+^ T lymphocyte counts changed significantly over time in group A (p_F_ = 0.0012, lower at day 21 pi compared with days 3, 70 and 98 pi, p_D_ < 0.05). **B** CD8^+^ T lymphocyte counts changed significantly over time in group A (p_F_ = 0.0002; lower at day 21 pi compared with days 3, 70 and 98 pi, p_D_ < 0.05). **C** CD5^+^ T lymphocyte counts changed significant over time in group A (p_F_ < 0.0002, lower at day 21 pi compared with days 3, 70 and 98 pi, p_D_ < 0.05). **D** CD4^+^/CD8^+^ ratio changed significantly over time in group A (p_F_ = 0.0002, lower at days 14 and 21 pi compared with day 0 and day 98 pi, p_D_ < 0.05). **E** B lymphocyte counts changed significantly over time in group A (p_F_ = 0.0103, lower at day 21 pi compared with day 3 pi, p_D_ < 0.05). **F** Activated CD4^+^ T lymphocyte counts changed significantly over time in group A (p_F_ < 0.0001, higher at days 3, 14 and 21 pi when compared with days 42, 56, 70 and 98 pi, p_D_ < 0.05) and group B (p_F_ = 0.0249, no significance in the posttest).

In the passively immunized cats, all analyzed lymphocyte subsets [CD5^+^, CD4^+^ and CD8^+^ T lymphocytes, activated CD4^+^ T lymphocytes (CD4^+^CD25^+^) and B lymphocytes (CD45R/B220^+^)], showed a significant change over time (p_F_ < 0.05). In the control cats, a significant change during the course of infection was only observed for the activated CD4^+^ T lymphocyte subset (p_F_ < 0.05). Specifically, cats in group A showed a significant decrease in the CD4^+^, CD8^+^ and CD5^+^ T lymphocyte counts, with the nadir reached at days 21, 21 and 28 pi, respectively, coinciding with the onset of peak bacteremia (CD4^+^, p_F_ = 0.0012; CD8^+^, p_F_ = 0.0002; CD5^+^, p_F_ < 0.0002, Figures 6A–C). The CD4^+^/CD8^+^ ratio was significantly increased at 2–3 weeks pi in the passively immunized cats (p_F_ = 0.0002, Figure 6D). Cats in group A showed a significant decrease in their B lymphocyte counts during maximal bacteremia, with the nadir reached at day 28 pi (p_F_ = 0.0103, Figure 6E), and the B lymphocyte counts were significantly higher at days 3, 14, 42 and 98 pi compared with cats in group B (p_MWU_ < 0.05; Figure 6E). When cat JHW3 was excluded from the analyses, the B lymphocyte counts were also higher in cats in group A compared to cats in group B, with significance reached at days 3 and 14 pi (P_MWU_ < 0.05). The activated CD4^+^ T lymphocyte subsets showed a somewhat different pattern than the other lymphocyte subsets in group A. The activated CD4^+^ T lymphocyte counts increased early during the course of infection into the onset of PCR-positivity and peak bacteremia, and then, they markedly decreased and reached a nadir at day 42 pi (p_F_ < 0.0001); this pattern was also present but less pronounced in group B (p_F_ = 0.0249, Figure 6F). Furthermore, compared with cats in group B, cats in group A showed significantly higher activated CD4^+^ T lymphocyte counts at day 14 pi (p_MWU_ = 0.0400) and significantly lower levels at day 42 pi (p_MWU_ = 0.0193; Figure 6F). When cat JHW3 was excluded from the analyses, a tendency for higher activated CD4^+^ T lymphocyte counts in cats in group A compared to group B was found at day 14 pi (P_MWU_ = 0.0539); the difference at day 42 pi was still significant (lower counts in cats in group A when compared to cats in group B; P_MWU_ = 0.024).

### Compared to control cats, passively immunized cats had higher bilirubin, total protein and globulin levels

Bilirubin concentrations significantly increased in both groups during the course of the experiment and peaked at day 49 pi (group A, p_F_ < 0.0001; group B, p_F_ = 0.0036; Additional file 1A). Bilirubin concentrations were lower in group A than in group B at days 0 and 14 pi (p_MWU_ = 0.04) but tended to be higher from day 28 until the end of the experiment, with significance reached at day 91 pi (p_MWU_ = 0.0080; Additional file 1A). The mean bilirubin concentrations in both groups remained within the reference interval (bilirubin concentration <3.5 μmol/L) throughout the study period; bilirubin concentrations exceeding the reference interval were observed in three cats in group A (DHR 1, day 49 pi, JHW1, day 49 pi; JHW5, day 56 pi, bilirubin concentrations, range 4.4–7.7 μmol/L). The total protein concentrations significantly changed over time in groups A (p_F_ < 0.0001) and B (p_F_ = 0.0003; Additional file 1B) and peaked at days 63 and 42 pi, respectively. Higher total protein concentrations were observed in cats in group A compared to cats in group B, with significance reached at days 21, 35 and 49 pi (p_MWU_ = 0.0400; Additional file 1B). When cat JHW3 was excluded from the analyses, total protein concentrations of group A were also higher compared to cats in group B with significance reached at days 3, 21 and 42 pi (P_MWU_ < 0.05). The mean total protein concentrations of both groups remained within the reference interval (total protein, reference interval for 16–30 weeks of age, 33–75 g/L) throughout the study period; total protein concentrations above the reference interval were observed in two cats in group A and one cat in group B (total protein concentrations between 76.2–78.6 g/L). The globulin concentrations significantly increased over time only in group A (p_F_ < 0.0001; Additional file 1C). Compared with cats in group B, cats in group A showed significantly higher globulin concentrations at days 21 and 35 pi (p_MWU_ < 0.05; Additional file 1C).

## Discussion

This is the first study to investigate the protective role of the humoral immune response against hemoplasma infection using a passive immunization experiment. Our study demonstrated that the passive transfer of antibodies from *Mhf*-recovered to naïve SPF cats does not prevent infection, high bacterial loads and the development of clinical signs following homologous challenge with *Mhf*. The passively immunized and control cats showed no differences in the onset and extent of bacteremia and anemia during the course of *Mhf* infection. Hicks et al. [28] recently documented that *Mhf*-recovered cats were protected from reinfection following re-challenge with a homologous *Mhf* isolate. Our study indicates that the presence of antibodies to *Mhf* cannot mediate protection against homologous challenge, but that cellular or innate immune mechanisms may be necessary to provide protection against *Mhf* infection. This is also in line with the study by Hicks et al. [28], which reported protection from *Mhf* reinfection in the absence of a pronounced Th2-type response in the cats after re-challenge. In the latter study, the protected cats showed no increase in anti-DnaK antibody and IL-10 mRNA levels following *Mhf* re-challenge.

The anti-DnaK antibody levels in the passively immunized cats were only higher in the first 2 weeks after transfusion than in the control cats, and they remained below the threshold for seropositivity up to day 28 pi. It is possible that the level of antibodies in the passively immunized cats following transfusion was not sufficiently high to prevent bacteremia. However, Hicks et al. [28] documented protection from *Mhf* although the antibody levels to DnaK did not rise after *Mhf* challenge infection and remained at rather low levels (mean log-transformed relative antibody level (RAL) < 1.2) when compared to the control cats with *de novoMhf* infection (peak mean RAL > 2.2). They concluded that if *Mhf*-specific antibodies had been responsible for immune protection in these cats, then low levels of anti-DnaK antibodies could be sufficient to provide protection against *Mhf* infection. Because the assay and the calculations used to quantify anti-DnaK antibodies in that study were different from those used in the present study, a direct comparison of antibody titers was not possible. Furthermore, it needs to be considered that antibodies with different epitope specificities that are not detected with current DnaK ELISA could mediate immune protection. However, assays to measure such antibodies are not available.

Infection enhancement, i.e. earlier onset of bacteremia and anemia, was recently documented in cats that had recovered from *CMt* infection and were re-challenged with *Mhf* [15]. All *CMt*-recovered cats were serologically positive before *Mhf* inoculation. The study suggested that the pre-existing antibodies against *CMt* might have had enhanced infection kinetics in the *CMt*-recovered cats [15]. Although infection kinetics were not different between the passively immunized and control cats in this study, our results still suggest that the transfer of antibodies from *Mhf*-recovered cats could potentially be harmful. The passively immunized cats showed a significant increase in RBC OF during the course of infection. This effect was not observed in the control cats, indicating that the increase was not simply due to a non-specific effect of plasma transfusion on RBC fragility. It can be hypothesized that the antibodies bound to the *Mhf* organisms attached to RBCs. RBC-bound antibodies have been found after primary *Mhf* infection [16, 39, 40] and can induce receptor-mediated RBC phagocytosis or the activation of the complement system [41]. In the present study, RBC-bound antibodies were not detected in the cats by Coombs test, but the sensitivity of this assay may be limited [42]. The bilirubin concentrations were also higher in the passively immunized versus control cats at some time points during infection. RBC destruction leads to the formation of free hemoglobin, which is processed by the mononuclear phagocytic system. During this process, bilirubin is formed, released in the blood stream and further metabolized and excreted by the liver. During pronounced RBC destruction, the capacity of the liver to metabolize bilirubin is overwhelmed, and hyperbilirubinemia occurs. Hyperbilirubinemia was observed in three cats of group A, although mean bilirubin concentrations in both groups remained within the reference interval. Nevertheless, the higher RBC OF values and occasionally higher bilirubin concentrations could point towards a more pronounced RBC destruction in the passively immunized cats following subsequent *Mhf* challenge exposure.

The passively immunized cats showed signs of a more pronounced immune response when compared to control cats. Cats of group A showed higher lymphocyte, monocyte and B lymphocyte counts and higher total protein and globulin concentrations than the control cats at several time points during the experiment. Furthermore, peak globulin concentrations coincided with peak anti-DnaK antibody levels in both groups. This result was also found in our recent study, where we documented polyclonal hypergammaglobulinemia during *Mhf* infection coinciding with high anti-DnaK antibodies [15]. We speculated that a large portion of the immune globulin pool was not hemoplasma-specific and was potentially due to a polyclonal B lymphocyte activation. The induction of autoreactive antibodies has recently been reported for the porcine hemoplasma *Mycoplasma suis* and is thought to be caused by the upregulation of B lymphocytes in response to changes to the RBC surface of the infected host [43].

It could be argued that the use of the homologous *Mhf* isolate for experimental challenge of the recipient cats that had been used to infect the plasma donor cats contributed to the more pronounced immune response in the passively immunized cats. This has been reported for other feline pathogens, i.e. feline coronavirus (FCoV) which can cause feline infectious peritonitis (FIP) [44]. During the pathogenesis of FIP, the virus targets macrophages and infection of these cells can be enhanced in the presence of antibodies (antibody-dependent enhancement, ADE) [45]. Takano et al. [44] showed that cats passively immunized with antibodies to serotype I FCoV showed an enhanced onset of disease following inoculation with the homologous serotype; this was not found when cats passively immunized with antibodies to serotype II FCoV were challenged with serotype I. The authors concluded that FCoV re-infection with the same serotype might induce ADE and could advance the development of FIP. Although ADE has also been suspected in bacterial infection [46], it is unknown whether it might play a role in the pathogenesis of feline hemoplasma infections [15]. Signs of infection enhancement have recently been documented in “*Candidatus* M. turicensis”-recovered, seropositive cats following a challenge with *Mhf* [15]. The study suggested that the presence of antibodies directed against *CMt* could enhance *Mhf* infection. However, to the best of our knowledge, disease enhancement after re-challenge with the same feline hemoplasma species has not been documented. In contrast, two studies showed that cats that had recovered from *Mhf* or *CMt* infection were protected from re-infection following re-challenge with the same hemoplasma species, respectively [27, 28]. Both studies had used an aliquot of the same *Mhf* or *CMt* isolate for re-infection that had been used for primary infection of the cats (personal communication, RHL).

The passively immunized cats showed a significant decrease in lymphocyte counts near the onset of peak bacteremia (around 3 weeks pi). This comprised decreases in absolute counts of CD4^+^ T lymphocytes, CD8^+^ T lymphocytes and B lymphocytes around three to 4 weeks pi, and was followed by an increase of the lymphocyte subsets until the end of the study. In two recent studies we reported a very similar pattern in the lymphocyte subsets following *Mhf* infection of naïve and *CMt*-recovered cats. The decrease was explained by the migration of the cells from the peripheral blood to the draining lymph nodes, where they become activated and proliferate [15, 28].

In the individual cats, the decrease in leukocyte, lymphocyte and neutrophil counts occurred just prior to the development of anemia and the onset of peak bacteremia. A similar pattern was also observed for the eosinophils, whereas the monocyte counts peaked at the onset of maximal anemia. A decrease in leukocyte and neutrophil counts could be caused by the increased consumption of these cells, which are involved in bacterial killing or, less likely, by reduced production due to undefined inhibitory factors [47]. Monocytosis could be found due to hemolysis [48] and was shown to be associated with *Mhf* infection in naturally infected cats [49]. We documented leukopenia and neutropenia, as well as decreased eosinophils, lymphocytes and increased monocytes, at times of peak *Mhf* bacteremia also in a recent study [15]. It is difficult to judge whether the decrease in the white blood cell subsets was due to bacterial replication and cell consumption or, conversely, whether the decrease in white blood cells facilitated bacterial replication.

Interestingly, one cat in group B (JHW3) stayed PCR-negative and seronegative during the 100-day follow-up period, but it developed bacteremia and seroconverted 154 days after *Mhf* inoculation. Although low-level bacteremia, below the detection limit of the PCR assay, cannot be completely excluded in this cat, we would expect a productive infection to result in seroconversion [20, 50]. Because all cats in this study were kept under strictly controlled hygienic conditions, vector-borne transmission between the cats can be excluded. However, all cats of group B were housed together and four out of five cats in group B tested *Mhf* PCR-positive at the time that bacteremia and seroconversion occurred in cat JHW3. Therefore, direct transmission between the cats, i.e., by aggressive interactions, seems most likely, although no obvious clinical signs of aggressive interactions were observed in the cats in this study at any time during the experiment. A direct transmission of *Mhf* through saliva seems unlikely because only low hemoplasma loads can be detected in saliva of *Mhf*-infected cats [22] and transmission by oral or subcutaneous inoculation of saliva has not been successful for another feline hemoplasma species, *CMt* [19].

The present study had some limitations. No masking was used during data collection and analysis. Furthermore, the relatively small groups size used in this study for animal welfare reasons could have masked significant differences between the passively immunized and control cats. However, as no protection but rather signs of infection enhancement were found in the passively immunized cats, the limited group size should not have affected the principal hypothesis addressed in this study.

The present study used an experimental set-up to address the protective role of passively transferred antibodies in *Mhf* infection. Experimental infection studies do have some inherent limitations when results are generalized to natural infections. However, to mirror the natural transmission of hemoplasmas most accurately, a published low-dose infection model was applied in this study [22]. The inoculated dose contained only 1000 copies of *Mhf*, which corresponds to approximately 0.05 μL of infectious blood from a naturally infected cat [22]. This small blood volume can easily be transmitted by blood-sucking arthropods or via aggressive interaction between cats—both of the latter transmission routes are assumed to be natural ways of transmission for feline hemoplasmas [17–19]. Therefore, the infectious dose applied to the cats should not be the reason for the lack of protection in the passively immunized cats.

Several measures were undertaken to ensure that the plasma used for passive immunization did not contain viable *Mhf* organisms that could transmit infection. The donor cats were tested weekly for at least eight consecutive weeks before the plasma was collected. All these samples and the plasma pool itself were tested in triplicate with a highly sensitive *Mhf*-specific qPCR assay [12] and revealed PCR-negative results. Furthermore, an aliquot of the plasma pool used for passive immunization was transfused into an adult SPF, the cat was followed for 23 weeks after transfusion and stayed PCR-negative.

In conclusion, passive immunization did not provide protection against experimental infection with *Mhf* but instead enhanced RBC fragility and was associated with a more pronounced immune response after infection. This result suggests that a humoral immune response in the absence of cellular immune mechanisms is insufficient to provide protection from *Mhf* infection. Potential vaccine candidates should include the induction of a cellular immune response against *Mhf*.

## Additional file

10.1186/s13567-016-0361-x **Bilirubin, total protein and globulin concentrations during the course of** ***Mhf*** **infection.** Mean (± SD) bilirubin (A), total protein (B), and globulin (C) concentrations during the 100-day post-inoculation period in group A (black dots, solid line) and group B (open triangles, dashed line). Cats were subcutaneously inoculated with *Mhf* at day 0. The onset of bacteremia in group A and B (day 7 pi for both groups) is indicated with a triangle. Boxes represent the time of peak bacteremia in cats in group A (grey box) and group B (dotted box). Statistically significant differences between groups A and B are indicated with asterisks (p_MWU_ < 0.05). (A) Bilirubin concentrations changed significantly over time in group A (p_F_ < 0.0001, higher at days 49, 56, 63, 70 and 91 pi compared with day 0 and days 7 and 14 pi, p_D_ < 0.05) and in group B (p_F_ = 0.0036, higher at day 49 pi compared with day 21 pi, p_D_ < 0.05). (B) The total protein concentrations significantly changed over time in cats in group A (p_F_ < 0.0001, higher at days 35, 42, 49, 56, 63, 70, 76 and 91 pi compared with day 0 and day 14 pi, p_D_ < 0.05) and in group B (p_F_ = 0.0003, higher at days 42 and 70 pi compared with day 0 and day 14 pi, p_D_ < 0.05). (C) The globulin concentrations changed significantly over time in cats in group A (p_F_ < 0.0001, higher at days 21, 42, 63, 70 and 76 pi compared with day 0 and day 14 pi, p_D_ < 0.05).

## References

[1] Messick JB (2004). Hemotrophic mycoplasmas (hemoplasmas): a review and new insights into pathogenic potential. Vet Clin Pathol.

[2] dos Santos AP, dos Santos RP, Biondo AW, Dora JM, Goldani LZ, de Oliveira ST, de Sa Guimaraes AM, Timenetsky J, de Morais HA, Gonzalez FH, Messick JB (2008). Hemoplasma infection in HIV-positive patient, Brazil. Emerg Infect Dis.

[3] Steer JA, Tasker S, Barker EN, Jensen J, Mitchell J, Stocki T, Chalker VJ, Hamon M (2011). A novel hemotropic Mycoplasma (hemoplasma) in a patient with hemolytic anemia and pyrexia. Clin Infect Dis.

[4] Tasker S, Peters IR, Mumford AD, Day MJ, Gruffydd-Jones TJ, Day S, Pretorius AM, Birtles RJ, Helps CR, Neimark H (2010). Investigation of human haemotropic Mycoplasma infections using a novel generic haemoplasma qPCR assay on blood samples and blood smears. J Med Microbiol.

[5] Sykes JE, Lindsay LL, Maggi RG, Breitschwerdt EB (2010). Human coinfection with *Bartonella henselae* and two hemotropic mycoplasma variants resembling *Mycoplasma ovis*. J Clin Microbiol.

[6] Hu Z, Yin J, Shen K, Kang W, Chen Q (2009). Outbreaks of hemotrophic mycoplasma infections in China. Emerg Infect Dis.

[7] Yuan CL, Liang AB, Yao CB, Yang ZB, Zhu JG, Cui L, Yu F, Zhu NY, Yang XW, Hua XG (2009). Prevalence of *Mycoplasma suis* (*Eperythrozoon suis*) infection in swine and swine-farm workers in Shanghai, China. Am J Vet Res.

[8] Jensen WA, Lappin MR, Kamkar S, Reagan WJ (2001). Use of a polymerase chain reaction assay to detect and differentiate two strains of *Haemobartonella felis* in naturally infected cats. Am J Vet Res.

[9] Foley JE, Pedersen NC (2001). ‘*Candidatus* Mycoplasma haemominutum’, a low-virulence epierythrocytic parasite of cats. Int J Syst Evol Microbiol.

[10] Willi B, Boretti FS, Cattori V, Tasker S, Meli ML, Reusch C, Lutz H, Hofmann-Lehmann R (2005). Identification, molecular characterization, and experimental transmission of a new hemoplasma isolate from a cat with hemolytic anemia in Switzerland. J Clin Microbiol.

[11] Willi B, Filoni C, Catao-Dias JL, Cattori V, Meli ML, Vargas A, Martinez F, Roelke ME, Ryser-Degiorgis MP, Leutenegger CM, Lutz H, Hofmann-Lehmann R (2007). Worldwide occurrence of feline hemoplasma infections in wild felid species. J Clin Microbiol.

[12] Willi B, Boretti FS, Baumgartner C, Tasker S, Wenger B, Cattori V, Meli ML, Reusch CE, Lutz H, Hofmann-Lehmann R (2006). Prevalence, risk factor analysis, and follow-up of infections caused by three feline hemoplasma species in cats in Switzerland. J Clin Microbiol.

[13] Santos AP, Conrado Fde O, Messick JB, Biondo AW, Oliveira ST, Guimaraes AM, Nascimento NC, Pedralli V, Lasta CS, Gonzalez FH (2014). Hemoplasma prevalence and hematological abnormalities associated with infection in three different cat populations from Southern Brazil. Rev Bras Parasitol Vet.

[14] Sykes JE, Terry JC, Lindsay LL, Owens SD (2008). Prevalences of various hemoplasma species among cats in the United States with possible hemoplasmosis. J Am Vet Med Assoc.

[15] Baumann J, Novacco M, Willi B, Riond B, Meli ML, Boretti FS, Hofmann-Lehmann R (2015). Lack of cross-protection against *Mycoplasma haemofelis* infection and signs of enhancement in “*Candidatus* Mycoplasma turicensis”-recovered cats. Vet Res.

[16] Tasker S, Peters IR, Papasouliotis K, Cue SM, Willi B, Hofmann-Lehmann R, Gruffydd-Jones TJ, Knowles TG, Day MJ, Helps CR (2009). Description of outcomes of experimental infection with feline haemoplasmas: copy numbers, haematology, Coombs’ testing and blood glucose concentrations. Vet Microbiol.

[17] Woods JE, Brewer MM, Hawley JR, Wisnewski N, Lappin MR (2005). Evaluation of experimental transmission of *Candidatus* Mycoplasma haemominutum and *Mycoplasma haemofelis* by *Ctenocephalides felis* to cats. Am J Vet Res.

[18] Woods JE, Wisnewski N, Lappin MR (2006). Attempted transmission of *Candidatus* Mycoplasma haemominutum and *Mycoplasma haemofelis* by feeding cats infected *Ctenocephalides felis*. Am J Vet Res.

[19] Museux K, Boretti FS, Willi B, Riond B, Hoelzle K, Hoelzle LE, Wittenbrink MM, Tasker S, Wengi N, Reusch CE, Lutz H, Hofmann-Lehmann R (2009). In vivo transmission studies of ‘*Candidatus* Mycoplasma turicensis’ in the domestic cat. Vet Res.

[20] Wolf-Jackel GA, Jackel C, Museux K, Hoelzle K, Tasker S, Lutz H, Hofmann-Lehmann R (2010). Identification, characterization, and application of a recombinant antigen for the serological investigation of feline hemotropic Mycoplasma infections. Clin Vaccine Immunol.

[21] Tasker S, Caney SM, Day MJ, Dean RS, Helps CR, Knowles TG, Lait PJ, Pinches MD, Gruffydd-Jones TJ (2006). Effect of chronic feline immunodeficiency infection, and efficacy of marbofloxacin treatment, on ‘*Candidatus* Mycoplasma haemominutum’ infection. Microbes Infect.

[22] Baumann J, Novacco M, Riond B, Boretti FS, Hofmann-Lehmann R (2013). Establishment and characterization of a low-dose *Mycoplasma haemofelis* infection model. Vet Microbiol.

[23] Tasker S, Caney SM, Day MJ, Dean RS, Helps CR, Knowles TG, Lait PJ, Pinches MD, Gruffydd-Jones TJ (2006). Effect of chronic FIV infection, and efficacy of marbofloxacin treatment, on *Mycoplasma haemofelis* infection. Vet Microbiol.

[24] Berent LM, Messick JB, Cooper SK (1998). Detection of *Haemobartonella felis* in cats with experimentally induced acute and chronic infections, using a polymerase chain reaction assay. Am J Vet Res.

[25] Dowers KL, Olver C, Radecki SV, Lappin MR (2002). Use of enrofloxacin for treatment of large-form *Haemobartonella felis* in experimentally infected cats. J Am Vet Med Assoc.

[26] Dowers KL, Tasker S, Radecki SV, Lappin MR (2009). Use of pradofloxacin to treat experimentally induced *Mycoplasma hemofelis* infection in cats. Am J Vet Res.

[27] Novacco M, Boretti FS, Franchini M, Riond B, Meli ML, Hofmann-Lehmann R (2012). Protection from reinfection in “*Candidatus* Mycoplasma turicensis”-infected cats and characterization of the immune response. Vet Res.

[28] Hicks CA, Willi B, Riond B, Novacco M, Meli ML, Stokes CR, Helps CR, Hofmann-Lehmann R, Tasker S (2015). Protective immunity against infection with *Mycoplasma haemofelis*. Clin Vaccine Immunol.

[29] Geret C, Riond B, Cattori V, Meli M, Hofmann-Lehmann R, Lutz H (2011). Housing and care of laboratory cats: from requirements to practice. Schweiz Arch Tierheilkd.

[30] Nesina S, Katrin Helfer-Hungerbuehler A, Riond B, Boretti FS, Willi B, Meli ML, Grest P, Hofmann-Lehmann R (2015). Retroviral DNA—the silent winner: blood transfusion containing latent feline leukemia provirus causes infection and disease in naive recipient cats. Retrovirology.

[31] Wardrop KJ, Weiss DJ, Wardrop KJ (2010). Clinical blood typing and cross-matching. Schalm’s veterinary hematology.

[32] Weissenbacher S, Riond B, Hofmann-Lehmann R, Lutz H (2011). Evaluation of a novel haematology analyser for use with feline blood. Vet J.

[33] von Dehn B (2014). Pediatric clinical pathology. Vet Clin North Am Small Anim Pract.

[34] Ackley CD, Cooper MD (1992). Characterization of a feline T-cell-specific monoclonal antibody reactive with a CD5-like molecule. Am J Vet Res.

[35] Ackley CD, Hoover EA, Cooper MD (1990). Identification of a CD4 homologue in the cat. Tissue Antigens.

[36] Monteith CE, Chelack BJ, Davis WC, Haines DM (1996). Identification of monoclonal antibodies for immunohistochemical staining of feline B lymphocytes in frozen and formalin-fixed paraffin-embedded tissues. Can J Vet Res.

[37] Vahlenkamp TW, Tompkins MB, Tompkins WA (2005). The role of CD4+CD25+ regulatory T cells in viral infections. Vet Immunol Immunopathol.

[38] Holznagel E, Hofmann-Lehmann R, Leutenegger CM, Allenspach K, Huettner S, Forster U, Niederer E, Joller H, Willett BJ, Hummel U, Rossi GL, Schupbach J, Lutz H (1998). The role of in vitro-induced lymphocyte apoptosis in feline immunodeficiency virus infection: correlation with different markers of disease progression. J Virol.

[39] Zulty JC, Kociba GJ (1990). Cold agglutinins in cats with haemobartonellosis. J Am Vet Med Assoc.

[40] Alleman AR, Pate MG, Harvey JW, Gaskin JM, Barbet AF (1999). Western immunoblot analysis of the antigens of *Haemobartonella felis* with sera from experimentally infected cats. J Clin Microbiol.

[41] Stokol T, Weiss JK, Wardrop KJ (2010). Immune-mediated anemias in the cat. Schalm’s veterinary hematology.

[42] Wardrop KJ (2005). The Coombs’ test in veterinary medicine: past, present, future. Vet Clin Pathol.

[43] Hoelzle LE, Zeder M, Felder KM, Hoelzle K (2014). Pathobiology of *Mycoplasma suis*. Vet J.

[44] Takano T, Kawakami C, Yamada S, Satoh R, Hohdatsu T (2008). Antibody-dependent enhancement occurs upon re-infection with the identical serotype virus in feline infectious peritonitis virus infection. J Vet Med Sci.

[45] Pedersen NC (2014). An update on feline infectious peritonitis: virology and immunopathogenesis. Vet J.

[46] Mahalingam S, Lidbury BA (2003). Antibody-dependent enhancement of infection: bacteria do it too. Trends Immunol.

[47] Razin S, Yogev D, Naot Y (1998). Molecular biology and pathogenicity of mycoplasmas. Microbiol Mol Biol Rev.

[48] Feldman BF, Ruehl WW (1984). Interpreting absolute WBC counts. Mod Vet Pract.

[49] Lobetti RG, Tasker S (2004). Diagnosis of feline haemoplasma infection using a real-time PCR assay. J S Afr Vet Assoc.

[50] Barker EN, Helps CR, Heesom KJ, Arthur CJ, Peters IR, Hofmann-Lehmann R, Tasker S (2010). Detection of humoral response using a recombinant heat shock protein 70, DnaK, of *Mycoplasma haemofelis* in experimentally and naturally hemoplasma-infected cats. Clin Vaccine Immunol.

