# Supplementary material for: Passive immunization does not provide protection against experimental infection with Mycoplasma haemofelis
Source: Vet Res. 2016 Aug 5;47:79. doi: 10.1186/s13567-016-0361-x (PMC4975915; doi:10.1186/s13567-016-0361-x)
Supplement: Supplementary file 1 — 10.1186/s13567-016-0361-x Bilirubin, total protein and globulin concentrations during the course of Mhf infection. Mean (± SD) bilirubin (A), total protein (B), and globulin (C) concentrations during the 100-day post-inoculation period in group A (black dots, solid line) and group B (open triangles, dashed line). Cats were subcutaneously inoculated with Mhf at day 0. The onset of bacteremia in group A and B (day 7 pi for both groups) is indicated with a triangle. Boxes represent the time of peak bacteremia in cats in group A (grey box) and group B (dotted box). Statistically significant differences between groups A and B are indicated with asterisks (pMWU < 0.05). (A) Bilirubin concentrations changed significantly over time in group A (pF < 0.0001, higher at days 49, 56, 63, 70 and 91 pi compared with day 0 and days 7 and 14 pi, pD < 0.05) and in group B (pF = 0.0036, higher at day 49 pi compared with day 21 pi, pD < 0.05). (B) The total protein concentrations significantly changed over time in cats in group A (pF < 0.0001, higher at days 35, 42, 49, 56, 63, 70, 76 and 91 pi compared with day 0 and day 14 pi, pD < 0.05) and in group B (pF = 0.0003, higher at days 42 and 70 pi compared with day 0 and day 14 pi, pD < 0.05). (C) The globulin concentrations changed significantly over time in cats in group A (pF < 0.0001, higher at days 21, 42, 63, 70 and 76 pi compared with day 0 and day 14 pi, pD < 0.05). [file 13567_2016_361_MOESM1_ESM.pptx]

## Slide 1
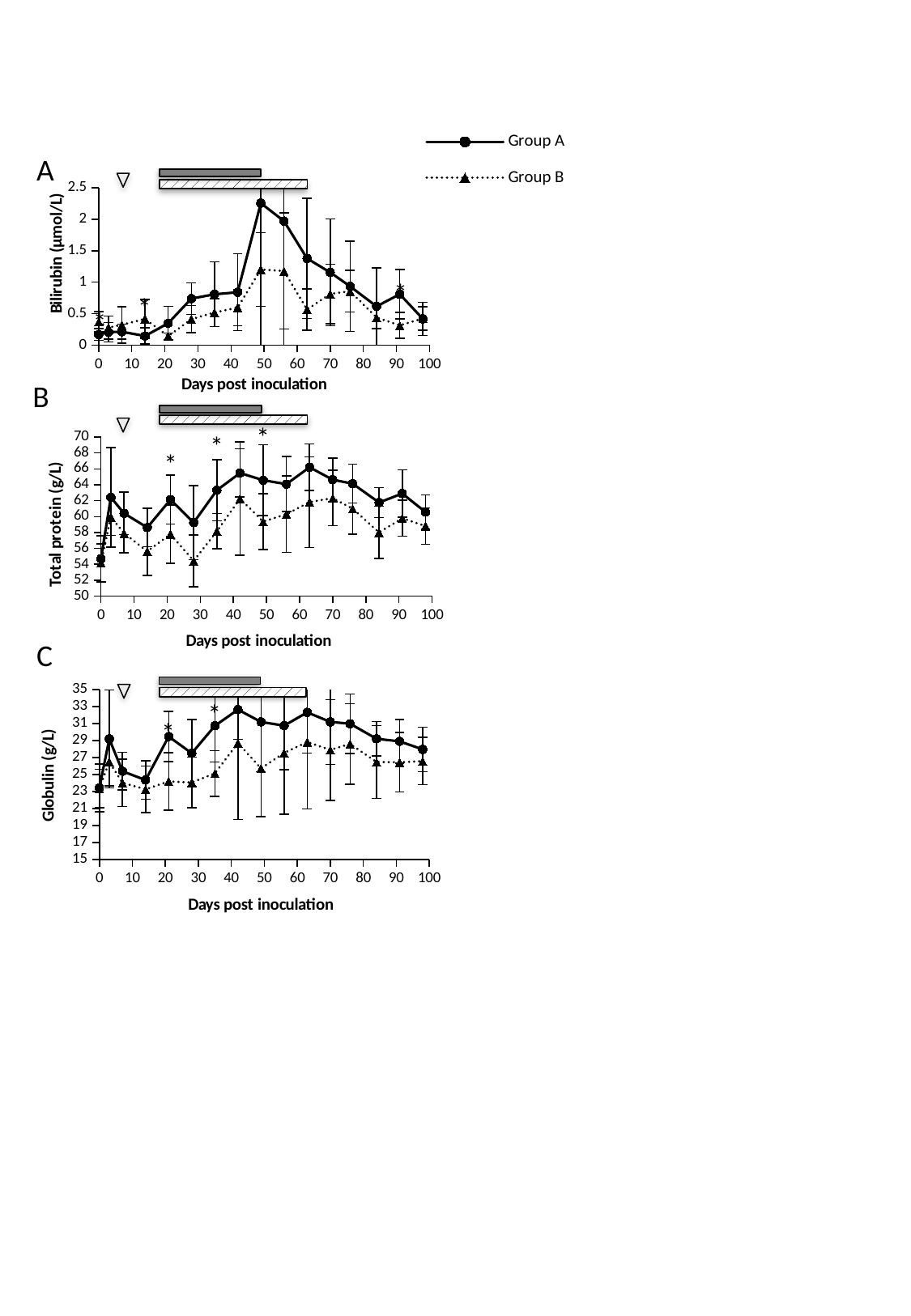

### Chart
| Category | Group A | Group B |
|---|---|---|A
*
*
*
B
*
### Chart
| Category | Group A | Group B |
|---|---|---|
*
*
C
### Chart
| Category | Group A | Group B |
|---|---|---|
*
*
